# Development and validation of a multimodal ultrasound-mammography model for predicting axillary lymph node metastasis in clinical Tl breast cancer

**DOI:** 10.1097/MD.0000000000048036

**Published:** 2026-03-20

**Authors:** Boyu Liang, Zhangjun Song, Furong Huang, Huxia Wang, Hongping Song

**Affiliations:** a Department of Ultrasound Medicine, Chinese First Affiliated Hospital of the People’s Liberation Army Air Force Medical University (Xijing Hospital), Xi’an, China; b Department of Breast Surgery, Yan’an People’s Hospital, Yan’an, China; c Department of Breast Disease Center, Shaanxi Provincial People’s Hospital, Xi’an, China.

**Keywords:** breast cancer, breast ultrasound, molybdenum target, nomogram

## Abstract

With the global expansion of breast cancer screening, early-stage detection, particularly clinical stage T1 breast cancer, has become increasingly common. Accurate prognosis for T1 cancer is closely linked to lymph node metastasis status. While breast ultrasound and mammography are standard for screening, they are suboptimal for axillary lymph node assessment. This study aimed to predict lymph node metastasis by analyzing primary tumor imaging characteristics. We retrospectively analyzed 148 patients with pathologically confirmed stage T1 breast cancer. Multivariate logistic regression identified independent risk factors for lymph node metastasis from breast ultrasound and mammography features. Subsequently, a line graph model was established to predict axillary lymph node metastasis. A predictive nomogram was developed and validated, demonstrating good discrimination with area under the receiver operating characteristic curve of 0.78 (training set) and 0.72 (validation set). The model also showed favorable calibration and clinical utility via decision curve analysis. In conclusion, this model can provide risk stratification for clinical T1-stage breast cancer axillary lymph node metastasis, which to some extent aids clinical decision-making, thereby reducing missed diagnoses and unnecessary invasive procedures. However, further prospective validation is still required.

## 1. Introduction

As the leading threat to women’s health worldwide, breast cancer has shown a progressive increase in incidence over the past few decades. Traditionally, the peak age of onset falls within the 50 to 70 year range.^[1]^ However, recent epidemiological data reveal a worrying trend toward younger populations. According to statistics from the National Cancer Institute,^[2]^ the incidence of breast cancer among women under 40 years of age exhibited a substantial increase of 18% between 2000 and 2020, with approximately 15% of these cases being clinically classified as T1 stage (tumor diameter ≤ 2 cm).A study demonstrated^[3]^ that the probability of axillary lymph node metastasis in clinical stage T1 breast cancer is 12%, with tumor diameter showing a strong correlation to the risk of axillary lymph node metastasis. However, existing clinical guidelines and predictive models are typically developed in cohorts encompassing all T-staging categories. May not adequately capture the subtle predictive features specific to the smaller tumor size and unique biological context of cT1 disease. Therefore, developing a dedicated prediction tool for this distinct subgroup is clinically imperative to enable more personalized and less invasive axillary management.

Preoperative assessment of lymph node metastasis directly informs the surgical approach. Sentinel lymph node biopsy (SLNB) is the current gold standard for evaluating axillary lymph node status. Compared with traditional axillary lymph node dissection (ALND), SLNB significantly reduces postoperative complications^[4]^ (e.g., lowering the incidence of lymphedema from approximately 25–5%). However, SLNB has notable limitations, including a false-negative rate of 5% to 10%,^[5]^ which may lead to disease underestimation and undertreatment. Furthermore, while about 70% of patients with positive SLNB undergo ALND, recent studies suggest that those with a low metastatic burden (1–2 micrometastases) may not benefit from ALND, as the survival advantage must be weighed against the risk of complications.^[6]^ Therefore, developing noninvasive and accurate tools for preoperative prediction of lymph node metastasis is crucial for optimizing individualized treatment and promoting the rational allocation of medical resources.

Our primary objective was to develop and internally validate a multimodal ultrasound–mammography prediction model (nomogram) for axillary lymph node metastasis in clinical T1 breast cancer. This model aims to predict the risk of lymph node metastasis in clinical stage T1 breast cancer to support precise surgical decision-making.

## 2. Materials and methods

### 2.1. General information

**Figure 1.**
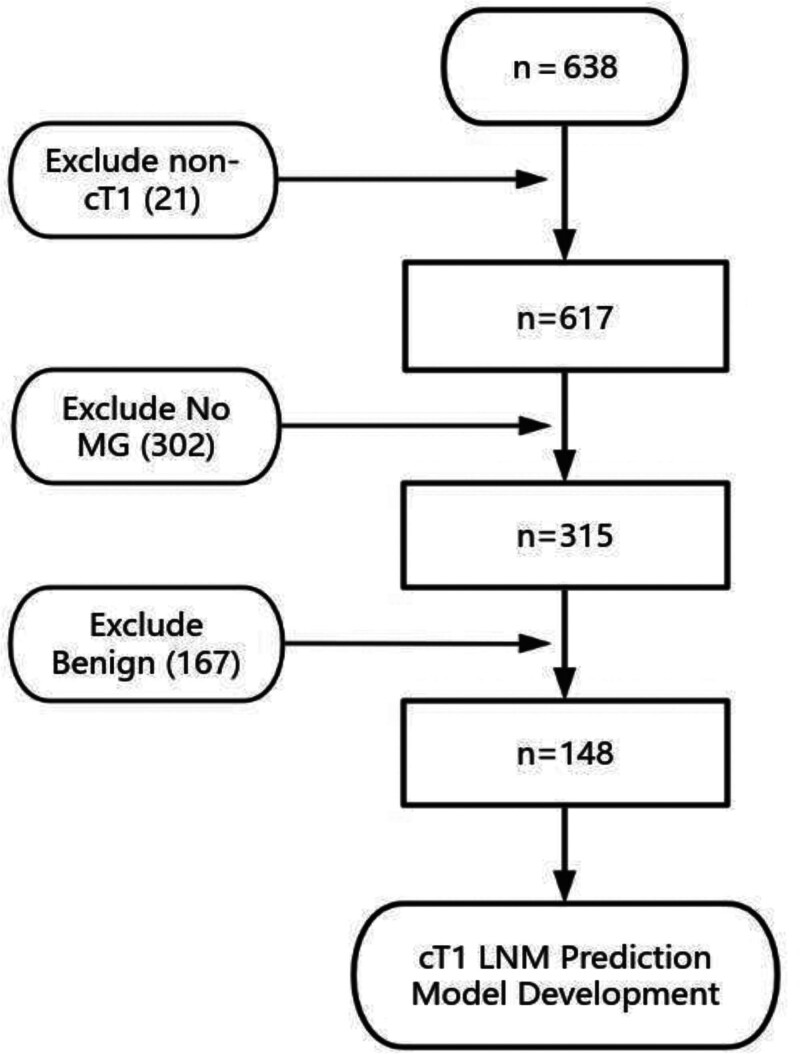
Flow chart of the included research subjects.

A total of 638 patients with clinical T1-stage breast lesion (according to the 8th edition of the American Joint Committee on Cancer guidelines). Clinical T1 tumors were defined as invasive carcinomas measuring ≤2 cm in the greatest dimension on preoperative imaging (ultrasound and mammography), who underwent parallel breast mass resection in the breast ward of Shaanxi Cancer Hospital from January 1, 2014 to December 31, 2017 were collected (Fig. 1).

*Inclusion criteria*: female; clinical stage Tl of the lesion; breast ultrasound (BUS) and mammography (MG) examinations were performed before surgery; pathological results are determined.

*Exclusion criteria*: prior history of radiotherapy or chemotherapy; incomplete visualization of the lesion on BUS; incomplete clinical or pathological data.

A total of 148 study cases were screened according to the inclusion and exclusion criteria.

### 2.2. Ethics approval and consent to participate

This study has been filed with the Medical Research Ethics Committee of Shaanxi Cancer Hospital. Ethics approval number: Medical Ethics Review (2024 Section) No. 21.

### 2.3. Grouping

To evaluate the generalizability of the model, the patient cohort was randomly divided into a training set and an internal validation set using R software (Version 4.4.1, R Foundation for Statistical Computing, Vienna, Austria, https://www.r-project.org), with 70% of the data allocated for training and the remaining 30% reserved for validation.

### 2.4. General clinical data collection

The following imaging features were collected from the study subjects: BUS characteristics, including lesion location, margin, shape, internal echogenicity, microcalcifications, capsule presence, and blood flow signals; and MG features, such as coarse calcifications, fine pleomorphic calcifications, architectural distortion, breast density, mass shape, margin characteristics (including spiculation), and associated findings.

### 2.5. BUS and MG image processing

BUS imaging features in both the training and validation sets were independently reviewed by a physician with over 5 years of experience in BUS. MG imaging features were assessed by a separate physician with >5 years of specialization in mammography. Pathological diagnoses, obtained via needle biopsy or surgical resection, were retrieved from the Department of Pathology of the participating cancer hospital. Based on the latest Breast Imaging-Reporting and Data System criteria,^[7]^ pathological outcomes were classified as either benign or malignant. Precancerous lesions, such as atypical ductal hyperplasia or atypical lobular hyperplasia, were excluded from the analysis. The radiologists who assessed the imaging features were blinded to the final histopathological results of axillary lymph node status during the feature extraction phase.

### 2.6. Pathological assessment

The primary outcome, axillary lymph node metastasis (ALNM), was determined by postoperative histopathological examination of SLNB or ALND specimens, which served as the gold standard. In accordance with American Joint Committee on Cancer 8th edition staging manual, a node-positive status was defined as the presence of either macrometastasis (>2 mm) or micrometastasis (deposit >0.2 mm but ≤2.0 mm). Isolated tumor cells (deposit size ≤ 0.2 mm) were recorded but classified as node-negative. Only metastatic involvement of axillary lymph nodes was considered for this outcome.

### 2.7. Imaging acquisition protocols

*BUS*: all breast and axillary ultrasound examinations were performed by experienced radiologists using standardized clinical protocols. Examinations were conducted using GE LOGIQ E9 ultrasound systems equipped with a linear array probe at a frequency of 9 to 15 MHz in breast mode. Imaging settings (including depth, gain, and focal zone) were optimized by the operator to ensure clear visualization of the target lesion and axillary anatomy.

*MG*: All patients underwent full-field digital mammography using Hologic Selenia Dimensions systems. Standard bilateral two-view mammography was obtained, including cranio caudal and mediolateral oblique views for each breast, following routine clinical practice.

### 2.8. Statistical methods

The training set (70% of the study cases randomized using R software) was used to develop the nomogram, and the validation set (30% of the study cases randomized using R software) was used to evaluate the nomogram. The Chi-square test was performed on the cases in the training set to determine the risk factors and included in the multivariate analysis, and the variables included in the multivariate analysis were regressed by the retrograde method to determine the independent predictors of clinical stage Tl breast cancer and lymph node metastasis. The nomogram is drawn based on the results of multivariate analysis, and then the nomogram is verified. Thousand bootstrap tests are used for internal validation to reduce model overfitting and provide more reliable prediction accuracy. The validation set data were used to validate the inner rows of the nomogram to evaluate its applicability and universality. The predictive accuracy and discrimination ability of nomograms are determined by calibration curves and the consistency index (C-index). Decision curve analysis (DCA) and clinical impact curve (CIC) were used to evaluate the clinical utility of nomograms. The difference was statistically significant with *P* < .05 (two-sided). Net benefit was calculated across a range of probability thresholds (p_t) from 20% to 80% using the standard formula: Net Benefit = (True Positives/N) ‐ (False Positives/N) × [p_t/(1–p_t)], where N is the total number of patients. Based on the clinical rationale threshold range considered by patients or clinicians between forgoing SLNB and undergoing the procedure, combined with relevant literature^[8]^ on the acceptability of regional lymph node metastasis risk, the selected threshold range was determined to be 0.2 to 0.8.

Statistical analysis and plotting using R software (version 4.4.1). The data were randomly divided using the “sample” function, the univariate analysis using SPSS version 26.0, and the multivariate logistic regression analysis using the “glm” function. Draw the nomogram using the “HMISC” package. The receiver operating characteristic (ROC) curve is plotted with the “pROC” package and the area under the ROC curve is measured. The Calibration Curves package is used to plot the calibration curves. Plotting DCA curves and CIC curves uses the “rmda” package.

## 3. Results

### 3.1. Univariate analysis results

The results (Table 1) showed that some BUS characteristics (boundary, morphology, and internal echo) and some MG characteristics (sand granular calcification and structural distortion). Spiculated margin were risk factors for lymph node metastasis in clinical stage Tl breast cancer (all *P* < .05), while some BUS characteristics (site, calcification, blood flow signal, and envelopes) and some MG features (coarse calcification, breast type, mass density, and the outline of the lump) were not associated with lymph node metastasis (all *P* > .05).

**Table 1:** Univariate analysis of risk factors for lymph node metastasis in clinical stage Tl breast cancer.

	Absence	Presence	Total	χ^2^	*P*-value
Total	81	67	148		
*Age*				0.03	.869
<50	41 (50.6)	33 (49.3)	74 (50)		
≥50	40 (49.4)	34 (50.7)	74 (50)		
*Molecular typing*				6.33	.176
Her-2 positive type	8 (9.9)	8 (11.9)	16 (10.8)		
Luminal A type	27 (33.3)	12 (17.9)	39 (26.4)		
Luminal B-type Her-2 (+)	13 (16)	16 (23.9)	29 (19.6)		
LuminalB-type Her-2 (‐)	21 (25.9)	24 (35.8)	45 (30.4)		
Triple-negative	12 (14.8)	7 (10.4)	19 (12.8)		
*ER*				2.45	.485
1+	31 (38.3)	26 (38.8)	57 (38.5)		
2+	15 (18.5)	10 (14.9)	25 (16.9)		
3+	13 (16)	17 (25.4)	30 (20.3)		
Negative	22 (27.2)	14 (20.9)	36 (24.3)		
*PR*				1.71	.634
1+	30 (37)	26 (38.8)	56 (37.8)		
2+	16 (19.8)	8 (11.9)	24 (16.2)		
3+	6 (7.4)	6 (9)	12 (8.1)		
Negative	29 (35.8)	27 (40.3)	56 (37.8)		
*Her-2*				3.08	.38
1+	15 (18.5)	13 (19.4)	28 (18.9)		
2+	11 (13.6)	16 (23.9)	27 (18.2)		
3+	15 (18.5)	12 (17.9)	27 (18.2)		
Negative	40 (49.4)	26 (38.8)	66 (44.6)		
*Ki-67*				3.164	.367
>10%≤30%	36 (44.4)	35 (52.2)	71 (48)		
>30%≤50%	8 (9.9)	7 (10.4)	15 (10.1)		
>50%	1 (1.2)	3 (4.5)	4 (2.7)		
≤10%	36 (44.4)	22 (32.8)	58 (39.2)		
*BUS variables*					
*Parts*				7.565	.182
Multicentric	0 (0)	2 (3)	2 (1.4)		
Upper inner quadrant	22 (27.2)	9 (13.4)	31 (20.9)		
Lower inner quadrant	4 (4.9)	3 (4.5)	7 (4.7)		
Nipple areola	5 (6.2)	4 (6)	9 (6.1)		
Upper outer quadrant	42 (51.9)	37 (55.2)	79 (53.4)		
Lower outer quadrant	8 (9.9)	12 (17.9)	20 (13.5)		
*Boundary*				7.9	.005
Unclear	45 (55.6)	52 (77.6)	97 (65.5)		
Clear	36 (44.4)	15 (22.4)	51 (34.5)		
*Morphology*				11.59	0
Irregular	55 (67.9)	61 (91)	116 (78.4)		
Regular	26 (32.1)	6 (9)	32 (21.6)		
*Internal echo*				9.58	.002
Uniform	46 (56.8)	21 (31.3)	67 (45.3)		
Inhomogeneous	35 (43.2)	46 (68.7)	81 (54.7)		
*Calcification*				0.93	.627
Macroscopic calcification	6 (7.4)	8 (11.9)	14 (9.5)		
Punctate calcification	19 (23.5)	16 (23.9)	35 (23.6)		
No calcification	56 (69.1)	43 (64.2)	99 (66.9)		
*Blood flow signals*				0.5	.777
Punctate blood flow signal	20 (24.7)	20 (29.9)	40 (27)		
No blood	33 (40.7)	25 (37.3)	58 (39.2)		
Abundant blood flow signal	28 (34.6)	22 (32.8)	50 (33.8)		
*Envelopes*				0.833	.361
No	80 (98.8)	67 (100)	147 (99.3)		
Yes	1 (1.2)	0 (0)	1 (0.7)		
*MG variables*					
Coarse calcification				0.68	.41
Without	58 (71.6)	47 (70.1)	105 (70.9)		
With	23 (28.4)	20 (29.9)	43 (29.1)		
*Sand granular calcification*				5.22	.022
Without	43 (53.1)	23 (34.3)	66 (44.6)		
With	38 (46.9)	44 (65.7)	82 (55.4)		
*Structural distortion*				4.89	.027
Without	61 (75.3)	39 (58.2)	100 (67.6)		
With	20 (24.7)	28 (41.8)	48 (32.4)		
*Type of mammary gland*				2.88	.718
Multi-fat type	11 (13.6)	11 (16.4)	22 (14.9)		
A small number of glandular types	8 (9.9)	7 (10.4)	15 (10.1)		
Low fat type	16 (19.8)	8 (11.9)	24 (16.2)		
Glandular type	8 (9.9)	6 (9)	14 (9.5)		
Fat type	9 (11.1)	5 (7.5)	14 (9.5)		
Dense type	29 (35.8)	30 (44.8)	59 (39.9)		
*Mass density*				2.282	.32
Equal density	12 (14.8)	5 (7.5)	17 (11.5)		
High density	64 (79)	59 (88.1)	123 (83.1)		
No density	5 (6.2)	3 (4.5)	8 (5.4)		
*The outline of the lump*				1.06	.589
Irregular	38 (46.9)	37 (55.2)	75 (50.7)		
Regular	38 (46.9)	27 (40.3)	65 (43.9)		
Indistinguishable outline	5 (6.2)	3 (4.5)	8 (5.4)		
*Spiculated margin*				11.87	0
Without	64 (79)	35 (52.2)	99 (66.9)		
With	17 (21)	32 (47.8)	49 (33.1)		

BUS = breast ultrasound, ER = estrogen receptor, MG = mammography, PR = progesterone receptor.

### 3.2. Results of multivariable analysis

Statistically significant variables after univariate analysis were included in the multivariate analysis, and multivariate logistic regression analysis was performed using the regression method (see Table 2). The results showed that there were differences between some BUS characteristics and some MG characteristics. Morphology: irregular vs regular (OR = 3.919, *P* < .05); internal echo: uniform vs inhomogeneous (OR = 2.234, *P* < .05); sandy-granular calcification: with vs without (OR = 2.319, *P* < .05); structural distortion: yes vs no (OR = 2.651, *P* < .05); spiculated margin: yes vs no (OR = 2.828, *P* < .05).

**Table 2:** Multivariate analysis of risk factors for lymph node metastasis in clinical stage Tl breast cancer.

Features	β	SE	OR	95% CI	*Z*	*P*
Boundary	0.632	0.418	1.882	0.83–4.27	1.514	.13
Morphology	1.366	0.532	3.919	1.381–11.118	2.567	.01
Internal echo	0.804	0.397	2.234	1.026–4.863	2.023	.043
Sandy granular calcification	0.841	0.395	2.319	1.069–5.03	2.13	.033
Structural distortion	0.975	0.416	2.651	1.173–5.991	2.344	.019
Spiculated margin	1.04	0.431	2.828	1.215–6.583	2.414	.016

### 3.3. Construct a nomogram to predict the risk of lymph node metastasis in clinical stage Tl breast cancer

**Figure 2.**
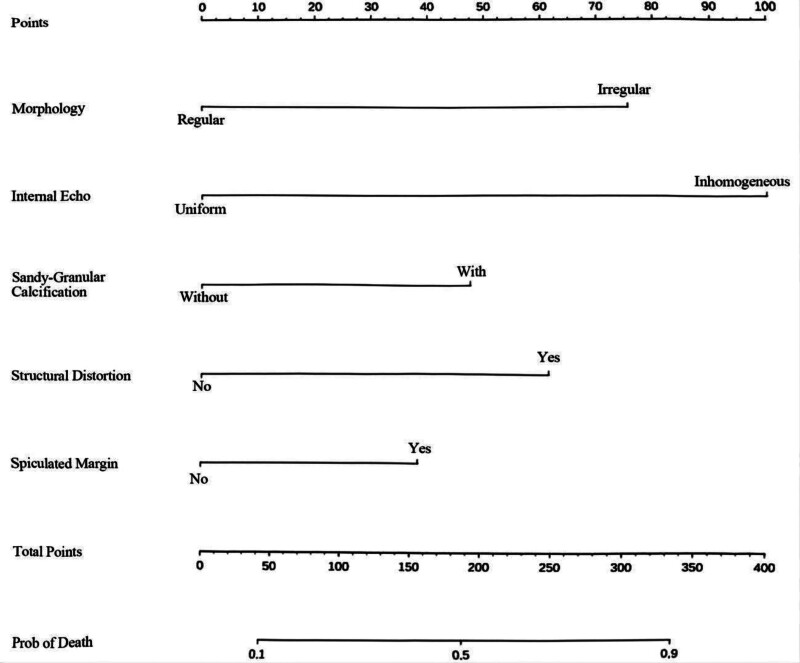
Nomogram of lymph node metastasis risk prediction for clinical stage Tl breast cancer.

Five independent predictors of lymph node metastasis in clinical T1 breast cancer were identified and incorporated into a predictive model, which was subsequently presented as a nomogram (Fig. 2). The nomogram indicates that internal echo homogeneity on BUS is the strongest predictor, followed by followed by whether the morphology in BUS is regular, whether there is structural distortion in MG, whether there is sandy granular calcification and whether there are spiculated margin.

### 3.4. Verification of nomograms

**Figure 3.**
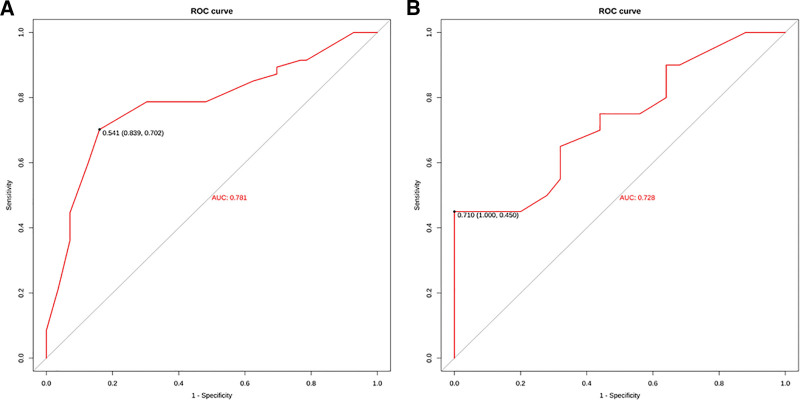
ROC curves of the training set (A) and validation set (B). ROC = receiver operating characteristic.

As shown in Figure 3, the area under the optimism-corrected ROC curve of the training set and the validation set is 0.78 (95% CI: 0.705, 0.857 n = 103) and 0.72 (95% CI: 0.646, 0.810 n = 45), respectively, indicating that the model has a good ability to predict whether there is metastasis in the lymph nodes of clinical stage T1 breast cancer.

### 3.5. Calibration of nomograms

**Figure 4.**
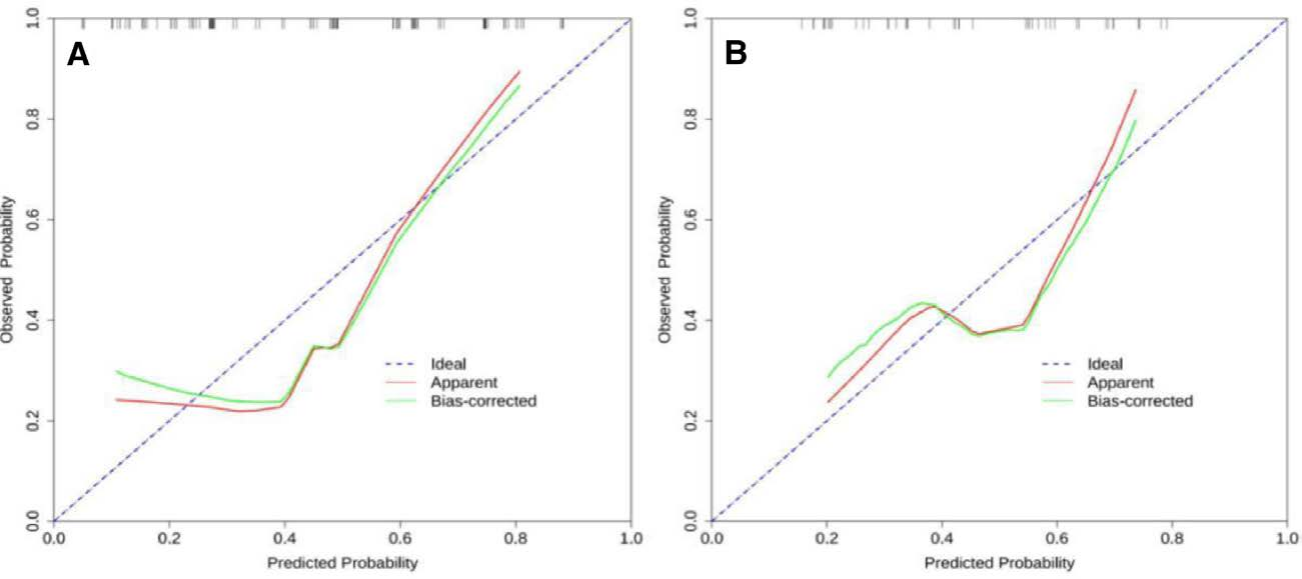
Calibration curves of the training set (A) and validation set (B).

The nomogram was internally verified by the 1000-time Bootstrap method, and the calibration curves of the training set and the validation set are shown in Figure 4. The relevant indicators of the calibration curve are shown in Table 3. The results showed that the fit between the predicted probability and the actual probability was good, that is, the consistency between the nomogram predicting the presence or absence of lymph node metastasis and the actual pathological results was good.

**Table 3:** Calibration curve correlation metrics.

Data set	Brier score	Intercept	Slope
Training set	0.1880703	‐2.157534e‐09 (approximately 0)	1.000000e+00
Validation set	0.2099476	‐0.08451555	0.81009028

### 3.6. DCA curve

**Figure 5.**
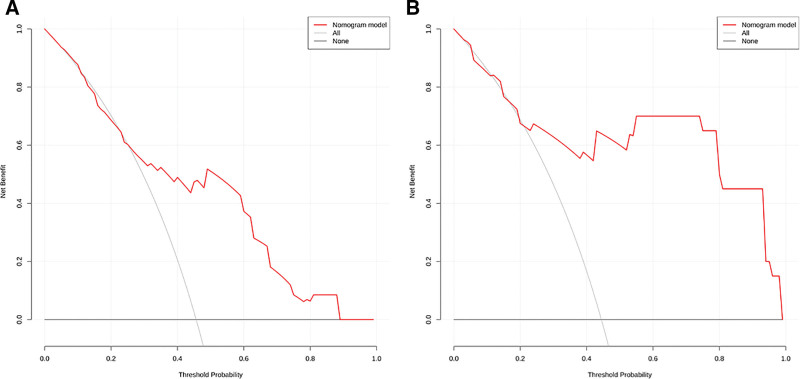
DCA curves of training set (A) and validation set (B). DCA = decision curve analysis.

**Figure 6.**
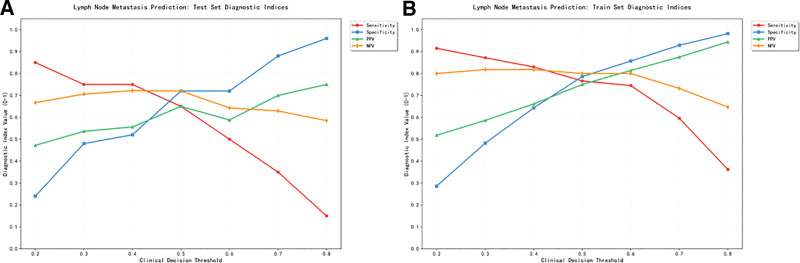
Line plots of relevant indicators for the training set (A) and validation set (B) at different clinical decision thresholds.

The results of this study show that when the threshold of the prediction model is 0.20 to 0.80, the decision curve is above the none line and the ALL line, indicating that the model showed a positive net benefit across threshold probabilities of approximately 20% to 80%. For clinical context, if the model were used at a threshold probability of 30% (implying a willingness to perform SLNB for *a* ≥ 30% predicted risk of ALNM) it would provide a net benefit equivalent to avoiding approximately 26 unnecessary SLNBs per 100 patients compared to the “biopsy all” strategy, while also resulting in the omission of about 6 additional positive lymph node cases (see Fig. 5, Tables 4 and 5). Line charts were plotted for different clinical decision thresholds (see Fig. 6), enabling clinicians to make trade-offs under varying conditions.

**Table 4:** Relevant indicators under different clinical threshold decisions (training set).

Threshold	TP	FP	FN	TN	Sensitivity	Specificity	PPV	NPV
0.2	43	40	4	16	0.915	0.286	0.518	0.8
0.3	41	29	6	27	0.872	0.482	0.586	0.818
0.4	39	20	8	36	0.83	0.643	0.661	0.818
0.5	36	12	11	44	0.766	0.786	0.75	0.8
0.6	35	8	12	48	0.745	0.857	0.814	0.8
0.7	28	4	19	52	0.596	0.929	0.875	0.732
0.8	17	1	30	55	0.362	0.982	0.944	0.647

**Table 5:** Relevant indicators under different clinical threshold decisions (validation set).

Threshold	TP	FP	FN	TN	Sensitivity	Specificity	PPV	NPV
0.2	17	19	3	6	0.85	0.24	0.472	0.667
0.3	15	13	5	12	0.75	0.48	0.536	0.706
0.4	15	12	5	13	0.75	0.52	0.556	0.722
0.5	13	7	7	18	0.65	0.72	0.65	0.72
0.6	10	7	10	18	0.5	0.72	0.588	0.643
0.7	7	3	13	22	0.35	0.88	0.7	0.629
0.8	3	1	17	24	0.15	0.96	0.75	0.585

### 3.7. CIC curve

**Figure 7.**
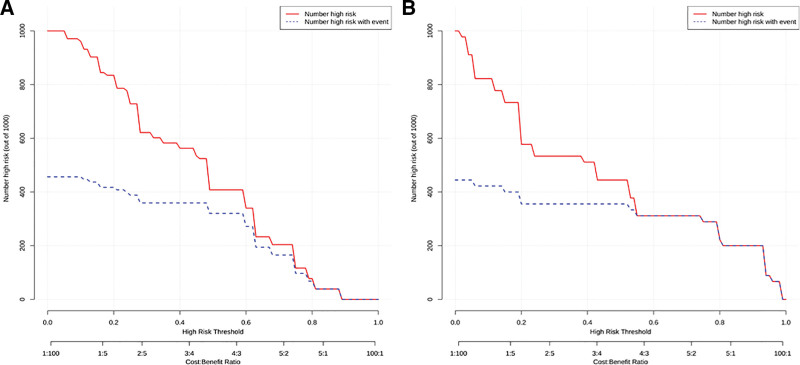
CIC curves of training set (A) and validation set (B). CIC = clinical impact curve.

The results of this study show that when the risk threshold is >0.5, the number of patients with high risk of lymph node metastasis predicted is basically similar to the actual number of patients with high risk of lymph node metastasis, indicating that the model has clinical practicability (see Fig. 7).

## 4. Discussion

Early prediction of lymph node metastasis in breast cancer is crucial for optimizing treatment decisions and improving patient outcomes. This study focused on clinical T1 breast cancer and examined the association between lymph node metastasis and imaging characteristics of the primary tumor using both BUS and MG. The results demonstrated that irregular morphology and heterogeneous internal echogenicity on BUS, as well as sandy granular calcification, structural distortion, and spiculated margins on MG, were independent risk factors for lymph node metastasis.

From a histological perspective, such morphological features are often associated with active fibroproliferative responses and stromal remodeling, which can create physical pathways for tumor cells to breach the basement membrane and invade adjacent lymphatic vessels.^[9]^ While certain sonographic features of the primary tumor (such as morphology and blood flow signals) have been linked to lymph node metastasis in other studies, the high-risk factors identified in our analysis align with part of those findings. Notably, blood flow signals did not emerge as a significant predictor of lymph node metastasis in this study, possibly due to the inclusion of only clinical T1 breast cancer patients, in whom detectable blood flow signals are often limited.

Furthermore, heterogeneous internal echogenicity on ultrasound reflects structural diversity within the tumor, which may indicate the presence of necrosis, microcalcifications, or alternating regions of high and low cellular density. This sonographic heterogeneity likely mirrors genomic instability within the tumor, a known driver of metastatic progression. Supporting this, a recent radiogenomic study demonstrated that heterogeneous echogenicity correlates with upregulation of epithelial–mesenchymal transition markers such as Vimentin and Snail.^[10]^ Epithelial–mesenchymal transition is a critical process through which tumor cells acquire migratory and invasive capabilities. In our study, internal echo heterogeneity was independently associated with lymph node metastasis risk (OR = 2.234, *P* = .043), suggesting its potential value as an ultrasonographic biomarker for predicting lymph node metastasis in clinical T1 breast cancer.

MG remains highly valuable in evaluating lymph node metastasis in early breast cancer. Sandy granular calcification have traditionally been considered a hallmark of ductal carcinoma in situ, yet their association with microinvasion has gained increasing attention. In this study, such calcifications (OR = 2.319, *P* = .033) were independently associated with lymph node metastasis in clinical T1 breast cancer, aligning with findings from a study by Yuan et al.^[11]^ Both results underscore the significant predictive value of sandy granular calcification on MG for lymph node metastasis.

On MG, infiltration of tumor cells into the surrounding stroma can induce a fibroproliferative response, which may manifest as local structural distortion or the formation of spiculated margins. Research by Cohen et al suggests that this fibroproliferative microenvironment activates the transformation of fibroblasts into cancer-associated fibroblasts via cytokine secretion (e.g., TGF-β and PDGF), thereby promoting lymphangiogenesis and tumor cell migration.^[12]^ In our study, structural distortion (OR = 2.651, *P* = .016) and spiculated margins (OR = 2.858, *P* = .019) were independently correlated with lymph node metastasis, indicating that fibroproliferative stromal reactions may serve as an important driver of lymphatic spread.

BUS and MG offer complementary insights into tumor biological behavior: MG reveals tumor-stromal interactions and microinvasive potential through features such as sandy granular calcification and structural distortion, whereas BUS more directly reflects tumor aggressiveness via morphological characteristics and internal echogenicity. In this study, the prediction model integrating both imaging modalities demonstrated favorable performance in assessing the risk of lymph node metastasis in clinical T1 breast cancer. Its clinical utility was further supported by DCA and CIC, providing a rational basis for stratified clinical decision-making within defined probability thresholds.

According to the model’s risk stratification, patients classified as low-risk (particularly those without calcifications or structural distortion on MG) could potentially avoid SLNB, thereby reducing the risk of complications such as lymphedema. Conversely, for patients presenting with irregular morphology on BUS along with sandy granular calcification on MG, more extensive lymph node dissection during surgery or intensified adjuvant therapy may be warranted. Furthermore, individuals exhibiting high-risk imaging features such as spiculated margins combined with heterogeneous internal echogenicity could be identified as having a elevated metastatic risk. For these patients, prioritizing neoadjuvant chemotherapy may help reduce nodal tumor burden. The clinical benefits of this imaging-based, risk-adapted management strategy require further validation in prospective studies.

This study has several important limitations that must be acknowledged when interpreting its findings. First, the model was developed and validated on a modest sample size from a single institution. Although internal validation via random split was performed, the limited sample may affect the stability of the estimated coefficients and the model’s statistical power. This underscores the necessity for external validation in larger, multicenter cohorts to confirm generalizability before any consideration of clinical application.

Second, our model is intentionally restricted to features from BUS and MG, reflecting a common preoperative imaging dataset. This design, however, omits other potentially powerful predictors. Notably, we did not integrate axillary ultrasound features, which are established factors in nodal metastasis. Future iterations of this model should aim to incorporate these elements to enhance predictive accuracy and clinical relevance.

Third, the exclusive use of data from a single tertiary cancer hospital may introduce spectrum bias. Our patient population likely represents a more selected or severe disease spectrum compared to community-screened populations. Consequently, the performance of this nomogram may vary in different clinical settings, and its generalizability requires direct testing in broader, more diverse populations.

Therefore, future research on clinical T1 breast cancer should adopt a prospective, multicenter design to systematically integrate multimodal radiomic features with genomic risk factors, thereby improving the predictive performance and clinical applicability of the model.

## 5. Conclusions

This study developed and internally validated a nomogram for predicting lymph node metastasis risk in patients with clinical T1 breast cancer. In this single-center retrospective study, a multimodal ultrasound–mammography nomogram showed moderate discrimination and calibration for predicting axillary lymph node metastasis in clinical T1 breast cancer. The model may support risk stratification, but external validation in larger, multicentre cohorts is needed before it can inform clinical decisions regarding axillary management.

## Author contributions

**Data curation:** Huxia Wang.

**Formal analysis:** Boyu Liang.

**Funding acquisition:** Zhangjun Song.

**Investigation:** Boyu Liang, Hongping Song.

**Methodology:** Hongping Song, Huxia Wang.

**Project administration:** Zhangjun Song.

**Software:** Furong Huang.

**Validation:** Boyu Liang.

**Writing – original draft:** Boyu Liang.

**Writing – review & editing:** Huxia Wang.
